# Multimodal Optical Imaging to Investigate Spatiotemporal Changes in Cerebrovascular Function in AUDA Treatment of Acute Ischemic Stroke

**DOI:** 10.3389/fncel.2021.655305

**Published:** 2021-06-03

**Authors:** Han-Lin Wang, Jia-Wei Chen, Shih-Hung Yang, Yu-Chun Lo, Han-Chi Pan, Yao-Wen Liang, Ching-Fu Wang, Yi Yang, Yun-Ting Kuo, Yi-Chen Lin, Chin-Yu Chou, Sheng-Huang Lin, You-Yin Chen

**Affiliations:** ^1^Department of Biomedical Engineering, National Yang Ming Chiao Tung University, Taipei, Taiwan; ^2^Department of Mechanical Engineering, National Cheng Kung University, Tainan, Taiwan; ^3^The Ph.D. Program for Neural Regenerative Medicine, College of Medical Science and Technology, Taipei Medical University, Taipei, Taiwan; ^4^National Laboratory Animal Center, Taipei, Taiwan; ^5^Department of Neurology, Hualien Tzu Chi Hospital, Buddhist Tzu Chi Medical Foundation, Hualien, Taiwan; ^6^Department of Neurology, School of Medicine, Tzu Chi University, Hualien, Taiwan

**Keywords:** multimodal optical imaging system, AUDA, neovascularization, cranial window, ischemic stroke, penumbra

## Abstract

Administration of 12-(3-adamantan-1-yl-ureido)-dodecanoic acid (AUDA) has been demonstrated to alleviate infarction following ischemic stroke. Reportedly, the main effect of AUDA is exerting anti-inflammation and neovascularization via the inhibition of soluble epoxide hydrolase. However, the major contribution of this anti-inflammation and neovascularization effect in the acute phase of stroke is not completely elucidated. To investigate the neuroprotective effects of AUDA in acute ischemic stroke, we combined laser speckle contrast imaging and optical intrinsic signal imaging techniques with the implantation of a lab-designed cranial window. Forepaw stimulation was applied to assess the functional changes via measuring cerebral metabolic rate of oxygen (CMRO_2_) that accompany neural activity. The rats that received AUDA in the acute phase of photothrombotic ischemia stroke showed a 30.5 ± 8.1% reduction in the ischemic core, 42.3 ± 15.1% reduction in the ischemic penumbra (*p* < 0.05), and 42.1 ± 4.6% increase of CMRO_2_ in response to forepaw stimulation at post-stroke day 1 (*p* < 0.05) compared with the control group (*N* = 10 for each group). Moreover, at post-stroke day 3, increased functional vascular density was observed in AUDA-treated rats (35.9 ± 1.9% higher than that in the control group, *p* < 0.05). At post-stroke day 7, a 105.4% ± 16.4% increase of astrocytes (*p* < 0.01), 30.0 ± 10.9% increase of neurons (*p* < 0.01), and 65.5 ± 15.0% decrease of microglia (*p* < 0.01) were observed in the penumbra region in AUDA-treated rats (*N* = 5 for each group). These results suggested that AUDA affects the anti-inflammation at the beginning of ischemic injury and restores neuronal metabolic rate of O_2_ and tissue viability. The neovascularization triggered by AUDA restored CBF and may contribute to ischemic infarction reduction at post-stroke day 3. Moreover, for long-term neuroprotection, astrocytes in the penumbra region may play an important role in protecting neurons from apoptotic injury.

## Introduction

Stroke is one of the cerebrovascular diseases with high mortality and associated with varied long-term disabilities worldwide (Benjamin et al., 2019). Notably, ischemic stroke accounts for approximately 87% of the cases of stroke. Ischemic stroke can lead to neuronal hypoxia and brain edema, further resulting in cell death (Lo et al., 2003). An intravenous thrombolytic agent is the only pharmacological therapeutic approach that can be administered in cases of acute phase of ischemic stroke; however, it has a limited application window of 4.5 h. With the time limitation and risky drawback of an increased brain hemorrhage, it only can be used in particular patients with balanced benefits (Whiteley et al., 2016). Nevertheless, administering a neuroprotective agent has been considered another potential treatment strategy in acute ischemic stroke therapy. Over 200 neuroprotective agents have been studied, but rarely applicable in human clinical trials (Lees et al., 2000; Sacco et al., 2001; Cheng et al., 2004; Lipton, 2004; Ginsberg, 2008; Ginsberg et al., 2013; Moretti et al., 2015; Razmara and Cramer, 2017).

The arachidonic acid epoxides—epoxyeicosatrienoic acids (EETs)—have been reported as an excellent therapeutic candidate for ischemic stroke treatment (Yuan et al., 2016; Geng et al., 2017). EETs have wide-ranging effects on brain protection, including anti-inflammatory, anti-apoptotic, vasodilatory, and pro-angiogenic reactions (Peng et al., 2002; Inceoglu et al., 2008; Terashvili et al., 2008; Iliff et al., 2009). However, EETs in the brain are rapidly metabolized by soluble epoxide hydrolase (sEH) into dihydroxyeicosatrienoic acids (DHETs), which are less bioactive than EETs in the brain (Yu et al., 2000; Wu et al., 2017; Chang et al., 2018). Therefore, sEH inhibition is currently considered an effective strategy for increasing EETs (Imig, 2006). The effective sEH inhibitor 12-(3-adamantan-1-yl-ureido) dodecanoic acid (AUDA) has been studied in the recent decades, with the research showing that it can reduce the cerebral infarct size of ischemic stroke (Dorrance et al., 2005; Qu et al., 2015; Hung et al., 2017; Chang et al., 2018; Yeh et al., 2019a,b). Several studies have demonstrated that AUDA protects brain injury against cerebral ischemia by regulating angiogenesis *in vivo* (Simpkins et al., 2009; Chang et al., 2018). Furthermore, AUDA administration reportedly alleviates microglial activation and attenuates inflammatory response following ischemic stroke (Liu et al., 2016; Chang et al., 2018). However, results from previous studies with discrete time points are unable to elucidate the role of AUDA in anti-inflammation and angiogenesis effects in the acute phase of ischemic stroke (Zhang et al., 2007; Tu et al., 2018).

In the present study, we used a photothrombotic ischemia (PTI) model in rats to investigate the protective mechanisms of AUDA administered in a time-course manner. We combined laser speckle contrast image (LSCI) technique and optical intrinsic signal imaging (OISI) technology (Zepeda et al., 2004; Lu et al., 2017) in a design with a setup of lenses and a color charge-coupled device (CCD) camera for simultaneously and independently measuring the cerebral blood flow and hemoglobin concentration. To assess the timeline of the neovascularization effect surrounding the lesion, the vessel density was quantified using speckle contrast images. Furthermore, the metabolic rate of oxygen (CMRO_2_) was calculated to evaluate the neural activity at different time points (Fox and Raichle, 1986; Coles et al., 2004; Vespa et al., 2005). The measured blood flow and hemoglobin concentration were used to recalculate CMRO_2_ by Fick’s Principle (Xu et al., 2009). To confirm that AUDA affects the neuroprotection and anti-inflammation reaction in the ischemic penumbra region, the brain sections were collected and double immunofluorescence staining was conducted to identify the viability of neurons, astrocytes, and microglia.

## Materials and Methods

### Animal Preparation and Grouping

Overall, 25 male adult Sprague–Dawley rats aged 8 weeks (weight: 300–350 g) were obtained from BioLASCO Taiwan Co., Ltd (Taipei, Taiwan). They were housed in the animal facility with well-controlled laboratory conditions and humidity (12/12 light/dark cycle with light at 7 AM; 20 ± 3°C) and were allowed free access to food and water. All experiments were performed in accordance with the approved guidelines and regulations of the Institutional Animal Care and Use Committee of the National Yang Ming University and Taipei Medical University.

To correlate to actual CBF, a flow phantom with different flow range values and dimensions compatible with rat brain vessels was used to calibrate the true flow speckle contrast acquired by our multimodal optical imaging system. We further evaluated the invasiveness of the lab-designed cranial window by assessing its effects on vascular density and vessel loss within the imaging windows over time (*N* = 5). For investigation of the neuroprotective effects of AUDA administration in acute ischemic stroke, we used a multimodal optical imaging system with varying characteristics, including LSCI and OISI through chronic cranial windows, to assess the functional changes in CBF and oxygen saturation that accompany neural activity. In total, 20 adult rats with photothrombotic stroke were randomly and equally divided into 2 groups—a control group (*N* = 10) and an AUDA-treated group (*N* = 10).

### Configuration of Multimodal Optical Imaging System

**FIGURE 1:**
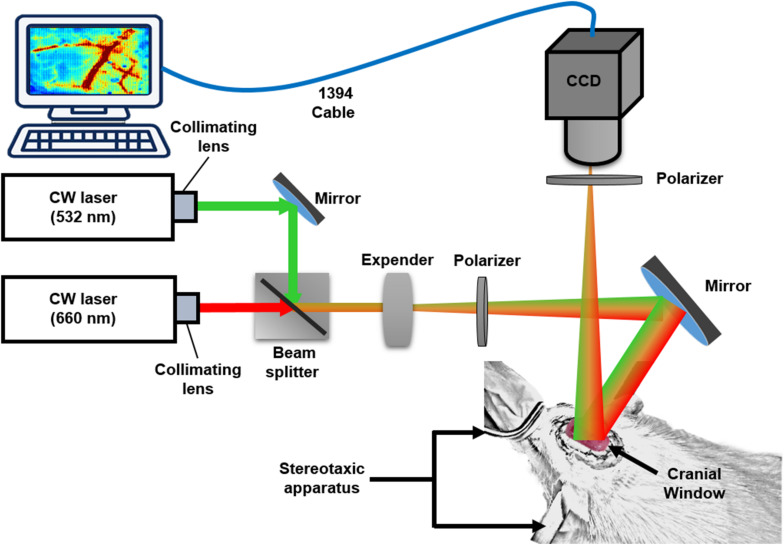
System setup. Multimodal optical imaging system consisting of two CW lasers (532 and 660 nm), a setup of lenses and the CCD camera. The CW 660-nm laser beam was reflected off moving red blood cells (RBCs) and produced speckle for LSCI analysis. The CW 532-nm laser beam was used to measure the absorbance difference by RBCs in vessels and surrounding tissues for OISI calculation. The optical setup was used to couple and expand the two laser beams and illuminate the rat cortex. Two polarizers were used to eliminate the noise reflected from the surface of the cortex. The CCD camera was positioned over the exposed area at an appropriate focal length, and the images were saved and transported to a personal computer via an IEEE 1394 interface for further data analysis. The rat was fixed in a custom-made stereotaxic frame with ear bars, and the surface of the rat brain was imaged through a lab-designed cranial window for LSCI and OISI analysis.

The multimodal optical imaging system, combining LSCI and OISI techniques, was used to simultaneously measure the changes in the dynamic blood flow and in hemoglobin oxygenation dynamics (Figure 1). A beam of continuous-wave (CW) laser (660 nm; 100 mW; RM-CW04-100, Unice E-O Service Inc., Taoyuan, Taiwan) and that of a coherent laser (532 nm; 100 mW; GM-XP02-100, Unice E-O Service Inc.) were collimated using a collimating lens that was mounted on each laser cavity. Thereafter, the 532-nm laser beam was reflected by the first mirror to a beam splitter (532 nm transmission rate < 2%; 532 nm transmission rate > 93%) that was placed between the 660-nm laser beam and expander and rotated at 45°. We adjusted the position and angle of the first mirror to combine the reflected 532-nm laser beam and transmitted 660-nm laser beam in the same beam path. The coupled laser beam was expanded using a plano-convex lens (f = 75 mm, LA1608-A, Thorlabs Inc., Newton, NJ, United States) to a size of approximately 40 mm × 30 mm and provided even illumination of the exposed area of the cortex. The illuminated area was imaged using a 16-bit CCD camera (1032 × 776 pixels; pixel size: 4.65 × 4.65 μm^2^, DR2-08S2M/C-EX-CS, Point Grey Research Inc., Richmond, BC, Canada) with Bayer filters (Wang et al., 2013) and an adjustable magnification lens (0.3 × to 1 ×, f/4.5 max) and a 2 × extender. The total magnification ratio was fixed at 2 × in the study, thus providing the field of view equal to the cranial window size (4 mm × 3 mm).

To satisfy the Nyquist sampling criterion and maximize the contrast of the imaged speckle pattern, the *f*-stop setting of the lens was changed to modulate the speckle size to five times the pixel pitch of the CCD camera (Kirkpatrick et al., 2008). A linear polarizer was placed before the CCD image collection lens to eliminate the noise that reflected from the tissue. The multimodal optical images were acquired at a frame rate of 15 Hz, and the exposure time was fixed at 10 ms for *in vivo* experiments. The entire system was controlled using a LabVIEW program (National Instruments Inc, Travis County, TX, United States).

### Data Process for LSCI

The speckle contrast images were obtained by quantifying the blurring of the speckle pattern that reflected from tissue and vessels over the integration time of the camera (Boas and Dunn, 2010). The speckle contrast map, *K*(*i, j*), is obtained by Eq. (1).

(1) $$K\,\left(i,\,j\right)=\,\frac{\sigma }{I}$$

where *K*(*i, j*) is the speckle contrast; (*i, j*) denotes the pixel coordinates in the image, and *σ* and *I* are the standard deviation of the intensity and mean intensity over a 5 × 5 pixel window.

The relationship between the speckle contrast and dynamic features of the speckle is an equation for *K*(*i, j*) based on the ratio of the correlation time τ_*c*_(*i, j*) of the backscattered light from the sample to the camera exposure time as in Eq. (2).

(2) $$K\,\left(i,\,j\right)=\,\sqrt{\frac{\tau_{c}\,\left(i,\,j\right)}{2\,T}\,\left\{1-\text{exp}\,\left(\frac{-2\,T}{\tau_{c}\,\left(i,\,j\right)}\right)\right\}}$$

where *T* is the exposure time of the camera (10 ms); and τ_*c*_(*i*,*j*) is the correlation time, which is assumed to be inversely proportional to the velocity of the scattering particles. Because the relationship between τ_*c*_(*i*,*j*) and the mean velocity of blood flow is known (Briers and Webster, 1996), by substituting $\tau_{c}\,\left(i,j\right)=\frac{\lambda }{2\,\pi \,V\,\left(i,j\right)}$, where λ is the optical wavelength of the coherent light source and *V*(*i, j*), into Eq. (2), we obtained Eq. (3) as follows:

(3) $$K\,{\left(i,\,j\right)}^{2}=\,\frac{\lambda }{4\,\pi \,V\,\left(i,\,j\right)\,T}\,\left\{1-\text{exp}\,\left(\frac{-4\,\pi \,V\,\left(i,\,j\right)\,T}{\lambda }\right)\right\}$$

in cases where $\text{exp}\,\left(\frac{-4\,\pi \,V\,\left(i,j\right)\,T}{\lambda }\right)$ approached 0 and thus the velocity of blood flow can be expressed as follows:

(4) $$V\,\left(i,j\right)\propto \frac{1}{\text{TK}\,{\left(i,j\right)}^{2}}\equiv \text{SFI}\,\left(i,j\right)$$

In our system, we utilized Eq. (4) to obtain the rCBF, as represented by the speckle flow index (SFI; Sigal et al., 2013; Li H. et al., 2014; Kazmi et al., 2015). To produce *K*^2^ -maps, 10 consecutive image frames were averaged in each pixel to reduce the noise.

The laser speckle flow images were calibrated with fresh arterial blood extract from male adult rats, following which it was confirmed that the change of *K*^2^ value and flow speed of flow phantom was linearly correlated in the range of 0.10–5.66 mm/s, thereby covering the range of true CBF velocity in the rat brain (Autio et al., 2011; Sato et al., 2017). The detailed description of calibration of the laser speckle flow imaging with a flow phantom is presented in Supplementary Note 1.

### Data Analysis for OISI

Changes in the hemoglobin concentration in cortical regions can be measured using the OISI technique (Morone et al., 2017). The changes in concentration of HbO and Hb—ΔHbO and ΔHb—were calculated according to the different absorbance of the tissue. The 660-nm laser beam coupled with the 532-nm laser beam in our optical system was used to generate the variations in diffuse reflectance (Reif et al., 2012; Wang et al., 2013). The Beer–Lambert model was used in the present study to estimate the changes in hemoglobin concentration (Jones et al., 2008; Qin et al., 2012; Reif et al., 2012), as presented in Eq. (5):

(5) $$\left[\begin{array}{c} \Delta \,\text{HbO}\,\left(i,\,j,\,t\right)\\ \Delta \,\text{Hb}\,\left(i,\,j,\,t\right) \end{array}\right]=\,{\left[\begin{array}{cc} \varepsilon_{\text{HbO}}^{\lambda_{1}} & \varepsilon_{\text{Hb}}^{\lambda_{1}}\\ \varepsilon_{\text{HbO}}^{\lambda_{2}} & \varepsilon_{\text{Hb}}^{\lambda_{2}} \end{array}\right]}^{-1}\,\left[\begin{array}{c} \frac{\ln\left(\frac{R_{\lambda_{1}}\,\left(i,\,j,\,0\right)}{R_{\lambda_{1}}\,\left(i,\,j,\,t\right)}\right)}{L_{\lambda_{1}}}\\ \frac{\ln\left(\frac{R_{\lambda_{2}}\,\left(i,\,j,\,0\right)}{R_{\lambda_{2}}\,\left(i,\,j,\,t\right)}\right)}{L_{\lambda_{2}}} \end{array}\right]$$

where ε is the molar extinction coefficient of the hemoglobin (cm^–1^/M), λ_*1*_ and λ_*2*_ are two different wavelengths—i.e., 532 and 660 nm, *R*_*λ1*_(*i, j, 0*) and *R*_*λ2*_(*i, j, 0*) are the baseline diffuse reflectance values from the first frame, *R*_*λ1*_(*i, j, t*) and *R*_*λ2*_(*i, j, t*) are the measured diffuse reflectance at each frame, and *L*_*λ_1_*_ and *L*_*λ_2_*_ are the differential path lengths of the light inside the medium, which equal to 0.04772 and 0.3534 cm, respectively. The change in total concentration of hemoglobin (Δ[HbT(*i, j, t*)]) was obtained by Δ[Hb(*i, j, t*)] + Δ[HbO(*i, j, t*)]. Every 10 reflectance frames were averaged together to reduce the noise on the reflectance maps. Data were analyzed off-line using custom-built MATLAB^®^ software (R2016b, MathWorks Inc., MA, United States).

### Implantation of Long-Term Cranial Windows

**FIGURE 2:**
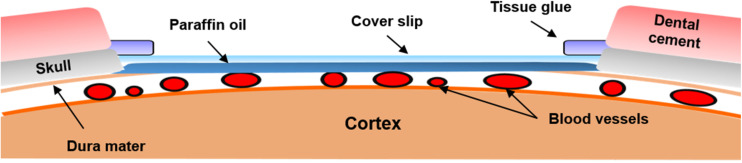
Illustration of the chronic cranial window. Schematics of the cranial window on the rat cortex. The cranial window was filled with paraffin oil and covered by a cover slip. To maintain the intracranial pressure, tissue glue was used to seal the gaps between the skull and cover slip. After the cranial window was set up, dental cement was placed around the cranial window to avoid damage to the cover slip.

A cranial window was set up to allow laser light access to the brain tissue and provide the ability to obtain clear images of the cortex. Each rat was anesthetized using a subcutaneous injection of 5 μg/kg dexmedetomidine hydrochloride (Dexdomitor^®^, Pfizer Inc., New York, NY, United States), which was commonly used in studies on cerebral blood flow and blood oxygenation responses evoked via forepaw stimulation (Bortel et al., 2019). The rats were then placed on a custom-made acrylic stereotaxic apparatus to reduce motion artifacts during the experiment. The skin of the scalp was removed from the skull to expose the bregma and the lambda as reference points. The location of the window was at the intersection of the middle cerebral artery (MCA) and anterior cerebral artery, which is associated with primary somatosensory cortex forelimb region (S1FL) and the primary somatosensory cortex hindlimb region (S1HL; anterior/posterior: -1 mm and medial/lateral: 1.5 mm; anterior/posterior: 3 mm; and medial/lateral: 4.5 mm to the bregma), according to a previous study (Pan et al., 2017). The window was drilled to a target size of 4 mm × 3 mm using a dental drill, and saline (Taiwan Biotech Co., Ltd., Taoyuan, Taiwan) was applied each 5 min for cooling. To obtain optical images from the cerebral cortex of rats for long-term observation, a lab-designed cranial window (Figure 2) was placed as follows. After the skull had been removed, the dura mater and arachnoid mater were carefully removed from the cerebral surface to expose the pia mater. Thereafter, the mineral oil (CAS#8020-83-5, Sigma-Aldrich, Saint Louis, MO, United States) was applied on the exposed brain surface in the window to avoid aqueous vapor appearing between the cortex and cranial window (Wahl et al., 1970; Navari et al., 1978; Kawamura et al., 1990). A cover slip was subsequently placed on the exposed brain tissue, and the edge of the cover slip was immediately glued onto the skull using tissue glue. After the cover slip was fixed, the cranial window was surrounded with dental cement to prevent the rats from scratching the window. After the cranial window was implanted, the implanted rats were housed individually, and they received post-operative care for 1 week for surgical recovery. According to previous studies, the cranial window setup can provide a high-resolution view for studying the activity of neuronal populations for long-term imaging. However, invasive surgery will induce injury and inflammation in the cortex over the long-term, thereby causing damage to the vessels and surrounding tissues (Xu et al., 2007; Dorand et al., 2014). To evaluate the stability of our lab-designed cranial window, the rat brain was imaged through the cranial window using our multimodal optical imaging system for 4 weeks. No variation of vascular density in the rat cortex under our lab-designed cranial window was observed over 4 weeks, indicating the high quality of our lab-designed cranial window (Li Y. et al., 2014). A detailed description of the vascular density calculation and the quantitative data analysis over the 4 weeks is provided in Supplementary Note 2.

### Photothrombotic Ischemic Stroke

After 1 week of recovery period since cranial window implantation, the rats underwent PTI induction. To produce an infarction in the S1FL brain area, a branch of the MCA within the S1FL on the right hemisphere (anterior/posterior: 1 mm; medial/lateral: 3.5 mm to the bregma) was targeted with a focal PTI stroke. The target vessel was selected based on its appearance and position within the anterior segment of the forelimb somatosensory map (Mohajerani et al., 2011). To induce embolism in the vessel, Rose Bengal photosensitizer (Na^+^ salt, R3877; Sigma-Aldrich Corp., St. Louis, MO, United States) was diluted to 10 mg/mL in HEPES-buffered saline and injected through the tail vein of the anesthetized rat at 30 mg/kg body weight.

**FIGURE 3:**
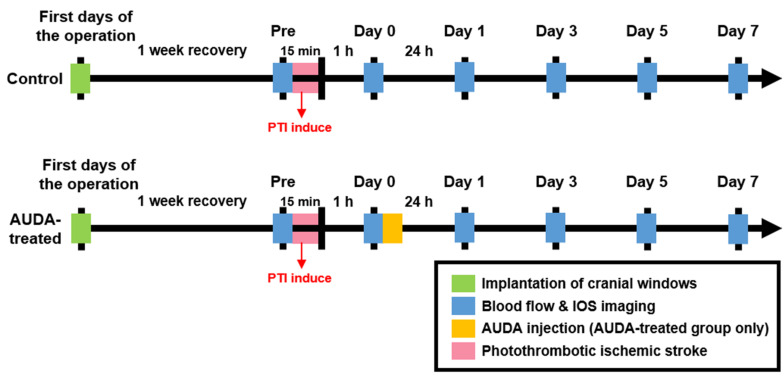
Experimental timeline for monitoring the effect of AUDA after PTI stroke. To monitor the effect of AUDA, ischemic stroke was induced by PTI in both groups for 15 min (red bar). After 1 h from PTI stroke, the rats in the AUDA-treated group received a single AUDA injection (yellow bar). After 1-week of recovery from the cranial window implantation operation (green bar), the images for LSCI and OISI analysis were recorded pre-PTI stroke and post-PTI stroke for 7 days (blue bar) in both groups.

After injecting the diluted Rose Bengal for 2 min, the dye diffused and entered the cerebral circulation. A surface arteriole in the S1FL area was then focally illuminated by a green-light laser (beam diameter: 1 mm; 532 nm, 5 mW, Unice E-O Service Inc., Taoyuan, Taiwan) for 15 min to induce infarction. After 1 h of PTI stroke, the rats in the control and AUDA-treated groups received an intraperitoneal injection of 0.9% saline or AUDA (10 mg/kg). The experimental timeline is shown in Figure 3.

### Definition of Ischemic Core and Penumbra With rCBF

The ischemic core was defined as the area with a decrease in rCBF of <20% of the value before any intervention, and the ischemic penumbra was defined as an area with a decrease in rCBF of approximately 20–50% (Fordsmann et al., 2019). The variation of rCBF was obtained using the equation *C*_*t*_(*i*,*j*) = *F*_*t*_(*i*,*j*)/*F*_0_, where *C*_*t*_(*i*,*j*) is the percentage change of rCBF on the *t*^*th*^ day after occlusion, *F*_*t*_(*i*,*j*) indicates the rCBF calculated by LSCI on *t*^*th*^ day after PTI stroke, and *F*_*0*_ represents the average of rCBF in the exposed cortex before PTI stroke. Consequently, two thresholds of rCBF reduction were selected in the present study: 20% for core definition and 50% for penumbra definition. Both areas were defined under the resting state of rats to avoid physiological interference. The zone sizes of both areas were calculated and converted proportionally to the area sizes in the images; they are displayed with dotted lines on the pseudo-color images.

### Measurement of Functional Vascular Density

We investigated neovascularization following PTI stroke using the functional vascular density (FVD) method to quantify changes in the vasculature (White et al., 2011). To calculate the FVD, over 30 consecutive speckle contrast images, which were the *K-map* images acquired from Eq. (1), were averaged and reconstructed into skeletonized vessel images based on the steps performed by Maragos and Schafer (1986). Accordingly, the intensity threshold in skeletonized vessel reconstruction was chosen until all the visible vessels on the speckle contrast image were reserved. Thereafter, the size threshold was determined to exclude the fractional noise from the image. The FVD was then measured by dividing the number of pixels of all skeletonized vessel segments by the total number of pixels of the cortex area through the cranial window, as shown in Eq. (6).

(6) $$\mathrm{FVD}=\frac{C}{n}$$

where *c* denotes the total number of pixels of the skeletonized vessel, and *n* denotes the total number of pixels on the cortex area through the cranial window. To assess the changes in the vascular density over time, the percentage change of FVD (ΔFVD%) was calculated by dividing the FVD value by the value of pre-strokes in the following equation:

(7) $$\Delta \,\text{FVD}\%\,=\,\frac{{\text{FVD}}_{t}-{\text{FVD}}_{\text{pre}}}{{\text{FVD}}_{\text{pre}}}$$

where FVD_*pre*_ denotes the FVD before PTI stroke and FVD*_*t*_* denotes the FVD on the *t*^*th*^ day with *t* = 0, 1, 3, 5, and 7.

### Calculation of Oxygen Consumption

To estimate the surrounding tissue metabolism of the infarct region, the cerebral metabolic rate of oxygen (CMRO_2_) was calculated from rCBF Eq. (4) and the hemoglobin concentration Eq. (5). Based on the calibration model from Fick’s principle, CMRO_2_ can be calculated using the following equation (Wise et al., 2013):

(8) $${\text{CMRO}}_{2}\,\left(i,j\right)=\left(\text{CBF}\,\left(i,j\right)\right)\,\left(\text{OEF}\,\left(i,j\right)\right)\,\left({\left[{\text{HbO}}_{2}\,\left(i,j\right)\right]}_{a}\right)$$

where CBF(*i*,*j*) is the absolute cerebral blood flow; OEF(*i*,*j*) is the oxygen extraction fraction by which the oxygen was removed from the vessels by the tissue, and [HbO_2_(*i*,*j*)]_*a*_ is the molar concentration in the arterioles. According to previous studies, (OEF(*i*,*j*))([HbO_2_(*i*,*j*)]_*a*_) is equivalent to the deoxygenated hemoglobin extracted by the tissue, as follows (Ghijsen et al., 2018; Wilson et al., 2019):

(9) $$\left(\text{OEF}\,\left(i,j\right)\right)\,\left({\left[{\text{HbO}}_{2}\,\left(i,j\right)\right]}_{a}\right)=4\,\left[\text{Hb}\,\left(i,j\right)\right]$$

where [Hb(*i*,*j*)] is the concentration of deoxyhemoglobin acquired from OISI.

Furthermore,*CBF*(*i*,*j*) in Eq. (8) was calculated from the rCBF acquired by using LSCI in the following equation:

(10) (CBF⁢(i,j))⁢=α⁢(SFI⁢(i,j))

where α is a conversion factor that correlates SFI(*i*,*j*) with the CBF(*i*,*j*), which equals the slope of the linear regression line obtained from the flow phantom experiment (see Supplementary Note 1). In previous studies and based on our flow phantom experiment, the results of the LSCI measurement revealed a high correlation and were consistent with flow index (*K*^2^) and absolute flow velocity (Winchester and Chou, 2004; Ambrus et al., 2016). Moreover, according to Eq. (4), the change in the SFI depends on the change in *K*^2^ with a fixed exposure time. Therefore, in the experimental setting, Eq. (8) can be re-written as follows:

(11) $${\text{CMRO}}_{2}\,\left(i,j\right)=4\,\alpha \,\left(\text{SFI}\,\left(i,j\right)\right)\,\left(\left[\text{Hb}\,\left(i,j\right)\right]\right)$$

To generate a CMRO_2_ map, 10 consecutive image frames were averaged to reduce the noise.

### Forepaw Electrical Stimulation

Forepaw stimulation was applied to evoke functional metabolic demands of oxygen in the S1FL brain area to quantify tissue activation. Two needle electrodes were subcutaneously inserted into the contralateral forepaw. The limb was stimulated by applying trains of rectangular pulses of 200 μs at a repetition rate of 9 Hz and constant current of 3 mA, which was supplied with a DS3 isolated current stimulator (Digitimer Ltd., Welwyn Garden, United Kingdom). The functional images were acquired during an OFF/ON block design forepaw electrical paradigm. Stimulation duration was 20 s followed by a resting period of 5 min to allow oxygen saturation to return to the resting state. Extra resting time (10 s) was used at the beginning of each scan. Therefore, each functional scan consisted of a 10-s pre-stimulus (OFF), 20-s stimulus (ON), 5-min inter-stimulus (OFF), 20-s stimulus (ON), and 5-min post-stimulus period (OFF). Here, the maxima value of CMRO_2_ averaged from two ON blocks was used to assess the stimulation-evoked hemodynamic response and oxygen consumption in the S1FL region. The area under the curve (AUC) of CMRO_2_ was calculated from the integral of the CMRO_2_ fractional change during the 20-s stimulus. The value in each stimulation period was divided by the baseline of the averaged 10-s pre-stimulus period.

### Immunohistochemical Staining

After 7 days of PTI stroke, 10 rats (control group: *N* = 5; AUDA-treated group: *N* = 5) were sacrificed; the rats underwent cardiac perfusion with 200 mL normal saline followed by 4% paraformaldehyde in phosphate-buffered saline (PBS) for 20 min. The brain was collected and post-fixed in 4% paraformaldehyde overnight and embedded in paraffin. To investigate the effect of AUDA administration in the ischemic lesions at post-stroke day 7, we aimed to label astrocytes, neurons, and inflammatory factors associated with tissue injury. DAPI was adopted to determine the number of nuclei and assess gross cell morphology. A total of 4 markers were acquired for multi-target immunofluorescence. However, labeling 4 markers on a brain section was unfeasible owing to the overlapped wavelength of emitted light; moreover, the multiple antibody titrations may impact surface antigen expression. To present the high-quality immunofluorescence images, we labeled 3 markers on a section with the fluorescent dyes. Total of 6 consecutive 3-μm-thick coronal brain sections were sampled from the PTI regions. Each paraffin wax-embedded brain section was cleaned in xylene and rehydrated by decreasing concentrations of ethanol. The six sections were then randomly incubated with 2 of the 3 primary antibodies: GFAP (1:500, Invitrogen, Camarillo, CA, United States), NeuN (1:500, Millipore, Billerica, MA, United States), or Iba1 (1: 500, Wako, Richmond, VA, United States) overnight at 4°C. Each brain section was then incubated with the appropriate fluorescence-conjugated secondary antibodies (Alexa Fluor 488- or 594-tagged, 1:500, Jackson ImmunoResearch Laboratories, West Grove, PA, United States) in PBS with 5% serum. All slides were then counterstained with DAPI in PBS (1:1000, Sigma-Aldrich, St. Louis, MO, United States). Brain sections were photographed using a confocal laser scanning microscope (LSM 880, Zeiss, Jena, Germany). To quantify the cells in the penumbra region, three sections per brain were independently and randomly selected at the margin of the injury site, and the number of immunofluorescence-positive cells was averaged for each rat. The image analysis was conducted using *ImageJ* software (National Institutes of Health, Bethesda, MD, United States).

### Quantitation of Infarct Volume With TTC Staining

To quantify the infarct volume of ischemic stroke, another 10 rats (control group: *N* = 5; AUDA-treated group: *N* = 5) were sacrificed at 7 days after PTI stroke and subsequently perfused with cold normal saline. Following perfusion, the brains were rapidly removed. Each brain was frozen at −20°C for 20 min, sliced into 2-mm coronal sections, and further stained with 2% 2, 3, 5-triphenyltetrazolium chloride (TTC; Sigma-Aldrich Corp., St. Louis, MO, United States) solution at 37°C for 20 min. After staining, the sections were washed with normal saline and fixed with 4% paraformaldehyde. The infarct volume was obtained by calculating the infarcted pixels in the brain sections (Benedek et al., 2006; Rupadevi et al., 2011) using *ImageJ*.

### Statistical Analysis

Significance tests among different days and within each group were evaluated using two-way analyses of variance (ANOVA), following which the Bonferroni *post-hoc* test was used to determine significant differences between testing groups. Non-parametric statistical analyses between two groups with small sample size (*N* < 6) were tested using two-sample Mann–Whitney *U*-tests. To investigate the effects of AUDA administration, we analyzed the size changes of the ischemic core and penumbra between the control and AUDA-treated groups on days 1, 3, 5, and 7 and assessed the variation within 7 days by performing a two-way ANOVA followed by Bonferroni *post-hoc* evaluation (*N* = 10 in each group). A probability value (*p*) of <0.05 was used as the criterion to determine statistical significance. The change of FVD between both groups was compared using the Mann–Whitney *U*-test (*N* = 10 in each group) with a significance threshold (*p*) of <0.05. To compare the neural activity in the S1FL area during forepaw stimulation, two-way ANOVA followed by Bonferroni *post-hoc* test with a significance level (*p*) of <0.05 was used to analyze the maximum CMRO_2_ changes and AUC of CMRO_2_ between the two groups within 7 days. Following the immunohistochemical staining, the comparison of NeuN^+^, GFAP^+^, and Iba1^+^ expression between the groups was explored using the Mann–Whitney *U*-test (*N* = 5 in each group) with a significance threshold (*p*) of <0.05. To quantify the treatment effect of AUDA between the control and AUDA-treated groups, the infarct volumes on day 7 were analyzed after TTC staining using a Mann–Whitney *U*-test (*N* = 5 in each group). Statistical significance was defined as *p* value of <0.05. All data were averaged and expressed as the mean ± standard error of the mean (SEM). SPSS version 26.0 (SPSS Inc., Chicago, IL, United States) was used for statistical analyses.

## Results

### AUDA Reduced Ischemic Penumbra Size and Prevented Ischemic Core Expansion

**FIGURE 4:**
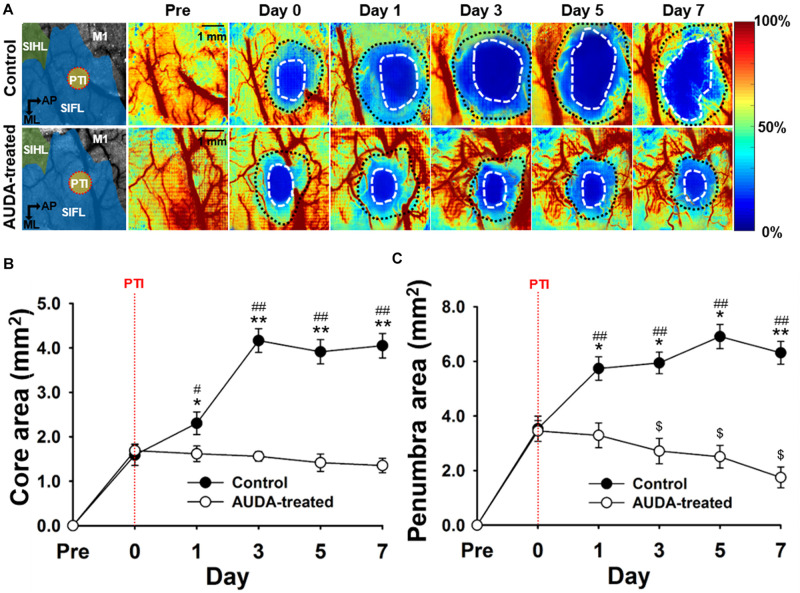
Comparison of ischemic core and penumbra region changes following PTI stroke. **(A)** Pseudo-color maps of the rCBF percentage change, based on the rCBF value before PTI stroke on day 0. The white dot circled area indicates the ischemic core with a threshold of 20% changes in rCBF. The area between the white dotted circle and black dotted circle contains the ischemic penumbra with a range of 20–50% changes in rCBF. The schematic diagrams of the brain area with S1HL, S1FL, and M1 area as well as the position of PTI induction are shown in the left panel. The scale bar is 1 mm. **(B)** The core size in the AUDA-treated group was significantly smaller than that of the control group from day 1 to day 7. Compared with the core size on day 0, the core size of the control group significantly increased on post-stroke day 1. The core size in the AUDA-treated group did not show significant changes following PTI stroke. Significant differences between groups are indicated by *p < 0.05 and **p < 0.01 and indicated by ^##^*p* < 0.01 compared with day 0 in both group (two-way ANOVA followed by Bonferroni *post-hoc* test). **(C)** The penumbra size in the AUDA-treated group was significantly reduced compared with that in the control group since post-stroke day 1. In addition, compared with day 0, a significant decrease in penumbra size was observed in the AUDA-treated group post-stroke day 3. Conversely, a significant increase in penumbra size was observed since post-PTI stroke day 0. Significant differences between groups are indicated by *p < 0.05 and **p < 0.01, by ^#^*p* < 0.05 and ^##^*p* < 0.01, and by ^$^*p* < 0.05 compared with day 0 in the control group (two-way ANOVA followed by Bonferroni *post-hoc* test). Data are presented as the mean ± SEM. *N* = 10 for each group. The red dotted line in both groups indicates the time of PTI stroke induction.

To assess the effects of AUDA on brain damages following ischemic stroke, the size of the ischemic penumbra and core areas were quantified by measuring rCBF. The temporal changes of ischemic penumbra and core were indicated in the pseudo-color images (Figure 4A). The results showed that the ischemic core of the both groups completely covered the PTI stroke induction site (Figure 4A, left panel, red dotted circle), and the size of both the ischemic core and penumbra areas were increased over time in the control group. We observed that the sizes of core were significantly increased in the control group from post-stroke day 1 (2.30 ± 0.25 mm^2^) and enlarged to 4.05 ± 0.27 mm^2^ at day 7. In the presence of AUDA, the core sizes were significantly decreased from post-stroke day 1 to day 7 compared with the respective controls by ∼66.7 ± 11.3% maximally (Figure 4B), and it indicated the deterioration of ischemic damage.

Further, the size of ischemic penumbra in the control group was significantly larger compared with that in the AUDA-treated group from day 1 (5.73 ± 0.42 mm^2^ for the control group and 3.28 ± 0.45 mm^2^ for the AUDA-treated group) to day 7 (6.31 ± 0.41 mm^2^ for the control group and 1.74 ± 0.38 mm^2^ for the AUDA-treated group) following PTI stroke. In addition, the size of penumbra of the AUDA-treated group was decreased at day 3 (2.71 ± 0.46 mm^2^) after PTI stroke compared with day 0 (3.44 ± 0.38 mm^2^). Conversely, in the control group, the penumbra area was increased by 0.8-fold on day 7 (6.31 ± 0.41 mm^2^) following PTI stroke compared with day 0 (3.53 ± 0.46 mm^2^; Figure 4C). These results suggested that AUDA decreased the PTI stroke-induced lesion size, including ischemic core and penumbra, and preserved the cerebral blood perfusion of the penumbra.

### AUDA Increased FVD in Ischemic Injury Area

**FIGURE 5:**
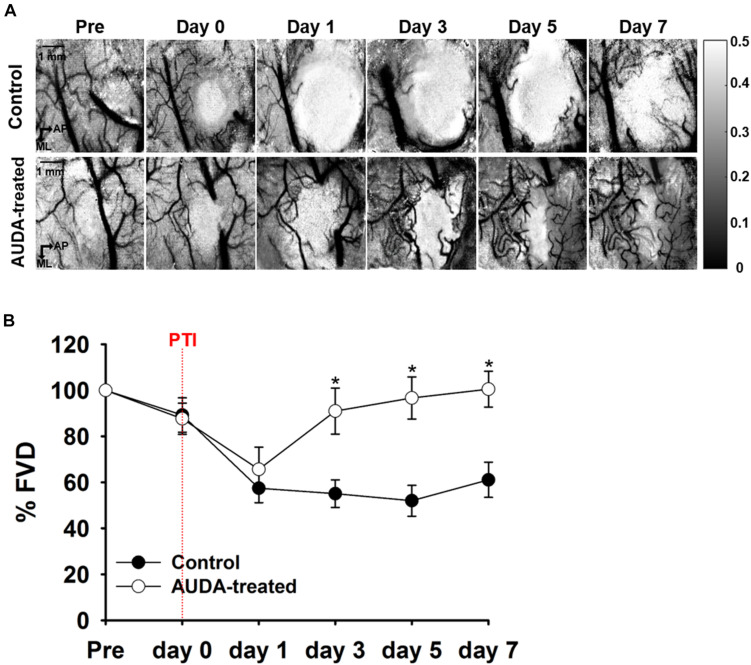
Comparison of %FVD in the observation window after PTI stroke. **(A)** *In-vivo* LSCI images over 7 days following stroke. The scale bar is 1 mm. **(B)** The %FVD was significantly higher in the AUDA-treated group than in the control group from post-PTI stroke day 3. Significant differences between groups are indicated by *p < 0.05 (Mann–Whitney *U*-test). Data are presented as the mean ± SEM. *N* = 10 for each group. The red dotted line indicates the time of PTI stroke induction.

To characterize the effects of AUDA administration on neovascularization following PTI stroke, the FVD was measured on each observation day. The representative speckle contrast images of control group in Figure 5A revealed that the signal of cerebral blood vessels was largely decreased following PTI stroke. Nevertheless, the AUDA-treated group showed evident new blood vessel development after stroke. The statistical results of FVD percentage change (%FVD) indicated that the vascular density of AUDA-treated group was significantly higher than that of the control group at post-PTI stroke day 3 day (%FVD = 0.91 ± 0.09% in the AUDA-treaded group and %FVD = 0.55 ± 0.05% in the control group; Figure 5B). These results demonstrated that the neovascularization in lesion area may begin at 3 days after AUDA administration.

### AUDA Treatment Restored Cerebral Metabolic Rate of Oxygen Consumption Surrounding Infarct Region

**FIGURE 6:**
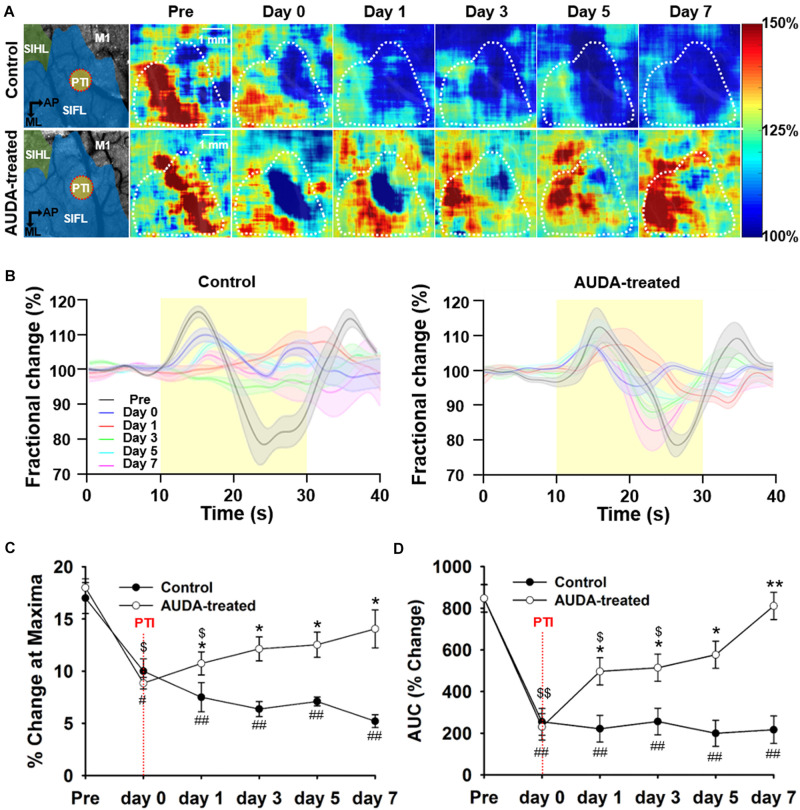
Comparison of CMRO_2_ changes during forepaw stimulation after PTI stroke. **(A)** Pseudo-color maps of maximum CMRO_2_ during forepaw stimulation. The white dotted circle represented the ROI in the S1FL area and was averaged for time statistical analysis, presented in **(C)**. The schematic diagrams of brain area with the S1HL, S1FL, and M1 areas, and the position of PTI induction are shown in the left panel. The scale bar is 1 mm. **(B)** The time series of fractional CMRO_2_ changes during forepaw stimulation and resting periods. The solid black line denotes the fractional CMRO_2_ changes pre-stroke; the blue, red, green, cyan, and magenta line indicate the fractional CMRO_2_ changes post-PTI stroke on days 0–7. The shadow error bar indicates the SEM across the subjects. **(C)** The maximum change of CMRO_2_ during forepaw stimulation was significantly higher in the AUDA-treated group than in the control group from post-PTI stroke day 1. In addition, the maximum response of CMRO_2_ was restored from day 3 in the AUDA-treat group compared with that observed pre-stroke. The restoration of maximum response of CMRO_2_ was not found in the control group post-PTI stroke. **(D)** The calculated area under the curve (AUC) indicates CMRO_2_ responses during forepaw stimulation. From day 1 to day 7, the calculated AUC in the AUDA-treated group was higher than that in the control group. Compared with pre-stroke, the calculated AUC was restored from day 5 in the AUDA-treated group. The restoration of CMRO_2_ responses was not observed in the control group post-PTI stroke. Significant differences between groups are indicated by *p < 0.05, **p < 0.01, indicated by ^#^*p* < 0.05, ^##^*p* < 0.01 compared with pre-stroke in the control group, and indicated by ^$^*p* < 0.05, ^$$^*p* < 0.01 compared with pre-stroke in the AUDA-treated group (two-way ANOVA followed by Bonferroni *post-hoc* test). Data are presented as the mean ± SEM. *N* = 10 for each group. The red dotted line indicates the time of PTI stroke induction.

To elucidate the beneficial effect of AUDA on PTI stroke, tissue oxidative metabolism evoked by forepaw electrical stimulation was quantified by measuring rCBF, Δ[Hb], and Δ[HbO]. Figure 6A shows the pseudo-color map of CMRO_2_ fractional change during forepaw stimulation at pre-stroke and post-stroke stages. The averaged CMRO_2_ fractional change that responded to forepaw stimulation is shown in Figure 6B. The response of CMRO_2_ to stimulation was elicited at the pre-stroke stage but was inhibited by PTI stroke on post-stroke day 0 for both groups. However, from post-stroke day 1 to day 7, responses of CMRO_2_ were observed in the AUDA-treated group but absent in the control group. The statistical results shown in Figure 6C indicate that the maxima change in CMRO_2_ was not restored in the control group in the days following stroke. However, AUDA administration recovered the blockage effect of controls by PTI stroke on CMRO_2_ changes, considering that the level of CMRO_2_ response at post-stroke day 1 was 10.74 ± 1.10%. The CMRO_2_ response was further enhanced at post-stroke day 7 by 14.05 ± 1.81%. Furthermore, in the AUDA-treated group, the level of stimulation-evoked oxidative metabolic response at post-stroke day 3 (12.14 ± 1.15%) was similar to that of the pre-stroke stage (18.00 ± 0.84%), suggesting the recovery of oxygen metabolism from ischemic damage. The CMRO_2_ change during forepaw stimulation was analyzed by calculating the AUC in Figure 6D. From this data, the AUC of the AUDA-treated group was found to be higher than the AUC of the control group since post-stroke day 1 (496.85 ± 65.44% of the AUDA-treated group and 221.55 ± 63.77% of the control group). Although the AUC at post-stroke day 5 (576.79 ± 65.14%) was similar to that in the pre-stroke stage (847.39 ± 67.25%) in the presence of AUDA, it largely decreased in the control group. Taken together, AUDA restored oxygen metabolism in the damaged brain cells. To properly evaluate the tissue oxidative metabolism during forepaw stimulation, we also provided the spatio-temporal maps and time course analysis of Δ[HbO], Δ[Hb], and Δ[HbT] to demonstrate the AUDA treatment effect on the PTI stroke model in Supplementary Note 3.

### AUDA Provides Neuron Protection by Modulating the Distribution of Astrocytes

**FIGURE 7:**
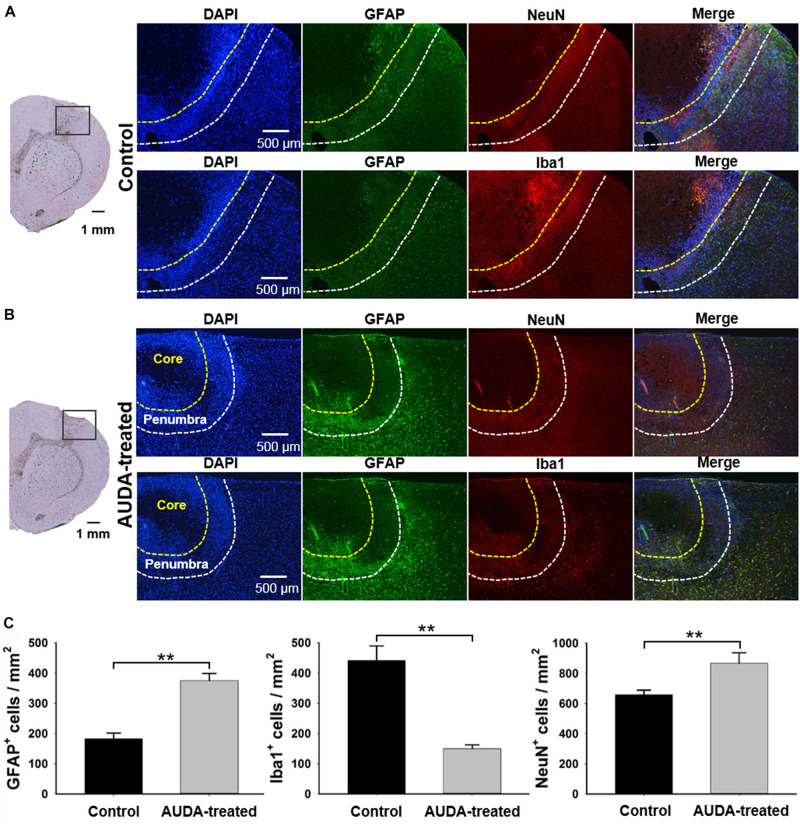
Comparison of NeuN^+^, GFAP^+^, and Iba1^+^ cell expression in ischemic core and penumbra at 7 days following PTI stroke. Representative images show double immunofluorescence staining of DAPI (blue), GFAP (green), NeuN (red), and Iba1 (red) in coronal sections under normal conditions at 7 days following PTI stroke. The observed region was selected within the boundary of the injury area under the bright field of the brain section, as shown in the left panel in the control group **(A)** and the AUDA-treated group **(B)**. The area of the ischemic core and penumbra are indicated by yellow dotted line and white dotted line, respectively, as established according to the expression intensity of NeuN^+^ cells. The 2 rows in a group represented 2 adjacent brain sections, containing all 4 markers. **(C)** The comparison of cell counts of NeuN^+^, GFAP^+^, and Iba1^+^ cells in the ischemic penumbra region in both groups. The density of GFAP^+^ cells and NeuN^+^ cells in the AUDA-treated group was significantly higher than that in the control group in the penumbra region. The number of Iba1^+^ cells in the AUDA-treated group was significantly lower than that in the control group in the penumbra region. The scale bar represents 500 μm in immunofluorescence images and represents 1 mm in the bright field of the brain sections. The symbol “^∗∗^” indicates significant differences (**p < 0.01, Mann–Whitney *U*-test). Data are presented as the mean ± SEM. *N* = 5 for each group.

To confirm the effects of AUDA in neuroprotection, double immunofluorescence staining of the neurons, astrocytes, and microglia was performed with NeuN, GFAP, and Iba1 antibodies at post-ischemic stroke day 7 (Figure 7). According to the immunofluorescence image of DAPI^+^ cells, two specific areas—the ischemic core and penumbra—were shown on the brain section. The ischemic core reflected weak immunoreactivity of DAPI^+^, indicating a major loss of neural cells. In addition, the penumbra contained dying neurons and increasing numbers of immunocytes, reflecting a massive density of DAPI^+^ cells. A significantly higher density of GFAP^+^ reactive astrocytes and NeuN^+^ cells were observed in the AUDA-treated group compared with the control group (NeuN^+^ cells: 658.3 ± 29.6 cells/mm^2^ in the control group and 866.4 ± 69.0 cells/mm^2^ in the AUDA-treated group, ^∗∗^*p* < 0.01, Mann–Whitney *U*-test; GFAP^+^ cells: 183.3 ± 18.0 cells/mm^2^ in the control group and 375.2 ± 23.5 cells/mm^2^ in the AUDA-treated group, ^∗∗^*p* < 0.01, Mann–Whitney *U*-test, *N* = 5 rats in each group). The activated microglia exhibited hypertrophic cell bodies; moreover, significantly lower levels of Iba1 could be detected in AUDA-treated animals compared with the untreated animals (Iba1^+^ cells: 441.6 ± 47.2 cells/mm^2^ in the control group and 150.7 ± 11.8 cells/mm^2^ in the AUDA-treated group, ^∗∗^*p* < 0.01, Mann–Whitney *U*-test, *N* = 5 rats in each group) Based on our results, the animals with AUDA administration following PTI stroke showed a higher survival rate of neurons; this may be attributable to a higher number of astrocytes in the penumbra and relatively milder brain inflammation following ischemic stroke.

### AUDA Reduced Infarct Volume in the PTI Stroke Model

**FIGURE 8:**
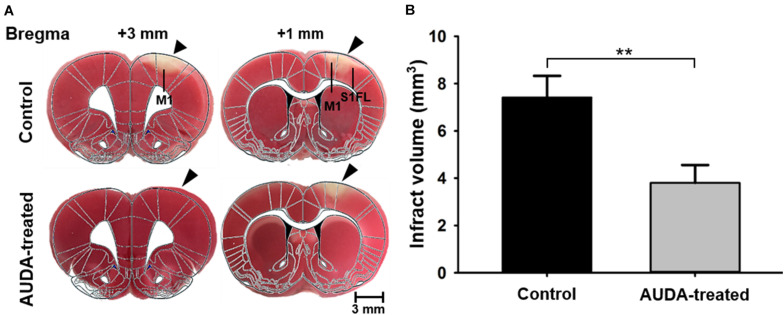
Comparison of infarct volume 7 days after PTI stroke. Analysis and quantification of infarct volume by TTC staining. **(A)** Coronal sections of ischemic rat brain at 1 and 3 mm anterior to the bregma. The red area is the normal area, and the light colored area is the infarct area, indicated by black arrowheads. **(B)** Statistical analysis of infarct volume. The infarct volume in the AUDA-treated group was significantly decreased compared with the control group on post-PTI stroke day 7. The scale bar is 3 mm. The symbol “^∗∗^” indicated significant differences (**p < 0.01, Mann–Whitney *U*-test, *N* = 5 for each group). Data are presented as the mean ± SEM.

To further evaluate the ischemic cortical lesion, we examined the infarct volume following PTI stroke by brain slicing and TTC staining on day 7 after the images were collected. Representative stained sections of each group are shown in Figure 8A. The unstained white color indicates the cortical infarcts induced by PTI stroke. The injury was predominant in the S1FL and primary motor cortex (M1) regions of the right hemisphere, at coordinates from 0.96 mm anterior to 3 mm anterior to the bregma at a depth of 1.4 mm from the cortical surface in the control group. The rats receiving AUDA injections in the acute phase of stroke showed a lower infarction volume (Figure 8B). The infarct volume in the AUDA-treated group was significantly reduced, to 51% of that of the control group (7.41 ± 0.9 mm^3^ in the AUDA-treated group and 3.8 ± 0.7 mm^3^ in the control group, ^∗∗^*p* < 0.01, Mann–Whitney *U*-test, *N* = 5 rats in each group).

## Discussion

In the present study, we combined LSCI and OISI techniques to investigate the effect of AUDA administration in the ischemic lesions in PTI stroke rats for 7 days. We observed that AUDA administration reduced the size of the ischemic penumbra and prevented the extension of the ischemic core in PTI rats. The angiogenesis possibly occurred earlier in AUDA-treated groups at post-PTI stroke day 3 compared with the control group. In addition, immunofluorescence staining was applied to explore the variety of different cells. The results indicated that AUDA may improve the astrocyte survival rate, thereby playing a role in rescuing the penumbra zone.

### LSCI and OISI Analysis for Pathological and Tissue Metabolism Studies in Ischemic Stroke After AUDA Treatment

Neuronal injury, death, and recovery following ischemic stroke is a time varied process. To study the dynamic effect of AUDA in acute ischemic stroke treatment, a measurement system simultaneously obtaining data on dynamic blood flow and changing hemoglobin oxygenation dynamics was required. Therefore, we used a system that combined LSCI and OISI in the study. Joseph P et al. have developed a diffuse optical tomography (DOT) technique for assessing tissue-averaged hemoglobin oxygen saturation (StO_2_) and rCBF (Culver et al., 2003). However, the reconstructed images of DOT are negatively impacted from poor spatial resolution owing to the diffuse nature of light in biological tissues (Dehghani et al., 2009). To solve spatial resolution, the LSCI technique with high spatial and temporal resolution (Takuwa et al., 2014; Chong et al., 2015; Ghijsen et al., 2018; Wilson et al., 2019) was adopted in combination with the OISI technique. Jones et al. first implemented a dual camera and dual light source LSCI and OISI system using a dichroic beam splitter (Jones et al., 2008). However, this system is rather restricted in its scalability. The majority of output illumination is lost through spectral filtering. Moreover, the use of dual cameras complicates alignment, results in different optical penetration depths, and limits co-registration of flow and oxygenation data. The use of mechanical moving components, such as the spinning source blocker, also leads to the same limitations, which were identified in another study (Du and Pan, 2011). This is a significant limitation in the fabrication of fast acquisition systems, particularly those requiring dynamic synchronization. Using our lab-designed multimodal optical imaging system, we were able to collect the flow rate and hemoglobin information from the same raw image with a high spatial resolution.

In addition to acquiring blood flow and hemoglobin information from the surface of the brain using the optical system, a PTI technique was employed to induce ischemia by illuminating the cortex with laser light. Although the MCAO method is considered more clinically relevant and has widely been used in AUDA studies (Dorrance et al., 2005; Qu et al., 2015; Chang et al., 2018; Yeh et al., 2019a,b), it is typically accompanied by high mortality and inconsistent lesion size variation (ranging from 5 to 50% of the cerebral hemisphere; Clark et al., 2019). Therefore, we adopted a photothrombotic method to reduce the control variable in the present study. Consequently, the design of a stable cranial window became an essential part of this study. Previous studies have offered valuable strategies for cranial window implantation, including the use of cover glass or polymer with the injection of a viscous substance between the brain and the covered substance (Goldey et al., 2014; Nishiyama et al., 2014; Park et al., 2015; Zuluaga-Ramirez et al., 2015; Heo et al., 2016). Although these methods are effective, successful retention of optical clarity with the removal of the dura mater is only achieved by a few of them. In the present study, the dura mater was differentiated from the cortex to provide clearer optical access to the brain. However, the injured dura mater could grow under the cranial window and impede optical transparency (Shih et al., 2012). Hence, we tightly adhered the margin of the dura mater to the cover glass with tissue glue, thereby maintaining intracranial pressure and preventing cerebral edema. Paraffin oil was applied between the brain and cover glass to push out the air and retain the moisture content inside the cranial window. This design of cranial window was able to maintain optical clarity over the course of the experiment and could provide a means of long-term observation for AUDA research.

### The Therapeutic Time Window of AUDA Treatment

Ischemic stroke is a dynamic event because the region of irreversible injury evolves gradually. However, the presence of a region surrounding the infarct area that was injured yet was salvageable (Memezawa et al., 1992) leads to the idea of a therapeutic time window (Schellinger and Kohrmann, 2012). In previous studies of neuroprotective agents in an ischemic stroke model, most acute stroke intervention trials-initiated therapy at the earliest after stroke onset. For instance, S-nitrosoglutathione was used to treat ischemic injury within 20 min of occlusion (Khan et al., 2006; Yeh et al., 2019a); puerarin, breviscapine, and EGb761 were shown to decrease the infarct volume of ischemic stroke following immediate injection (Zhang et al., 2012; Hongyun et al., 2017; Pengyue et al., 2017), and galangin was given intragastrically 15 min prior to experimental occlusion to maintain cortical blood flow (Li et al., 2012). However, the time from stroke onset is typically unclear in clinical situations, and a large proportion of patients arrive at a treatment center after the approved time limit for medication administration. Therefore, the therapeutic time window extension was one of the potential approaches to increase the probability of successful stroke treatment. Compared with the abovementioned neuroprotective agents, in our study, AUDA showed a therapeutic effect when administered as an intraperitoneal injection at 1 h after PTI stroke. A longer therapeutic time window for AUDA administration has been reported in recent studies (Yeh et al., 2019a,b). AUDA injected 2 h after MCA occlusion also improved behavioral outcomes and decreased infarct size. This research demonstrates the potential of AUDA for the treatment of ischemic injury over a prolonged therapeutic time window.

### AUDA Protects Against Ischemic Injury Possibly by Anti- inflammation and Neovascularization

Previous studies have indicated that AUDA modulates the vascular repair function (Dai et al., 2019) and promotes angiogenesis in the injured zone (Chen et al., 2016). Moreover, AUDA has been suggested to enhance blood flow and suppress local inflammatory responses (Dorrance et al., 2005). Although different neuroprotective effects of AUDA have been proposed, the dynamic mechanism of AUDA in treating acute ischemic stroke remains unknown. According to the results in our study, the effects of ischemic damage recovery by AUDA have two different mechanisms—enhancement of neovascularization and anti-inflammation. During the treatment of ischemic injury, an increase in blood flow is typically accompanied by an increase in the number of blood vessels through angiogenesis (Banai et al., 1994; Zhang et al., 2013). We observed that the border of the ischemic penumbra significantly reduced (rCBF = 20–50%) 3 days after AUDA treatment. Similarly, the FVD change across 1 week provided evidence of neovascularization in the lesion on day 3. The results reveal that the AUDA enhanced neovascularization earlier in the AUDA-treated group than in the control group after stroke. To investigate the functional recovery of ischemic stroke between the AUDA-treated and control groups, we compared oxidative consumption based on the CMRO_2_ changes that occurred during functional stimulation. Interestingly, the response to CMRO_2_ in the AUDA-treated group was significantly higher than the response of the control group on post-stroke day 1. The probable cause was attributed to the anti-inflammatory effect of AUDA exerted at the beginning of the ischemic stroke. Earlier studies have proposed that the neurons were protected against injury in the early phase of tissue damage (Morin et al., 2010; Liu et al., 2016; Dai et al., 2019; Yeh et al., 2019b); a similar finding was reported by Qu et al. (2015) that AUDA inhibited neuronal apoptosis in ischemic penumbra following the MCAO method. Furthermore, our study demonstrated that AUDA exerted neovascularization enhancement from post-ischemic stroke day 3. Two distinct mechanisms with different time points may explain the neuroprotective effect of AUDA in acute ischemic stroke.

### Effect of AUDA Administration on Glial Cells in Response to Ischemic Brain Injury

Glial cells, including astrocytes and microglia, were considered major mediators that act during cerebral inflammation. Previous studies have reported multiple brain cellular reactions following ischemic conditions in response to CNS insults. The phenomenon includes the expression of toxic inflammatory mediators produced by activated microglia (Alexandrova et al., 2004; Clausen et al., 2008), and enhancement of autophagy reactions (Wang et al., 2006) as well as astrogliosis and astrocyte hypertrophy (Sofroniew and Vinters, 2010). Microglia are the principal immune cells of the brain and rapidly respond to the pathophysiological changes in ischemic stroke (Gülke et al., 2018). Typically, activated microglia are considered a deleterious reaction in ischemic stroke owing to the acute expression of tumor necrosis factor-alpha (TNF-α) that aggravates the neurological deficit (Dawson et al., 1996; Barone et al., 1997; Clausen et al., 2008). Moreover, the microglia affected the release of reactive oxygen species, thereby damaging macromolecules, such as proteins and lipids, and leading to cell apoptosis and necrosis of the neurons (Zhang et al., 2016; Yang et al., 2018). Consecutively, the damaged cells lead to an extension of the ischemic core and penumbra region over time, as observed in the present study.

Furthermore, astrocytes play a key role in the regulation of neural development and growth as well as degeneration. Reactive astrogliosis is a general response of astrocytes in brain lesions as diverse as trauma or ischemia (Pekny and Nilsson, 2005). In ischemia studies, astrocytes have been observed to increase in number and size surrounding an ischemic infarction (Kajihara et al., 2001). In the penumbra induced by the MCAO method, AUDA reportedly protects the neurons from oxygen–glucose deprivation by stimulating the production of astrocyte-derived brain-derived neurotropic factor (Yuan et al., 2016). Within a few days after brain injury, a glial scar appears surrounding the necrotic brain tissue of the infarct that is generated by reactive astrocytes. Similar to scarring in other organs and tissues, the glial scar is a mechanism to protect and initiate the healing process in the nervous system (Sofroniew, 2009). In the acute phase of stroke, the glial barrier reportedly blockades the lesion area to prevent further infections and spread of cellular damage. Moreover, the glial barrier maintains ion and fluid balance in the extracellular environment and prevents excessive inflammatory and growth factor responses (Rolls et al., 2009). However, the ability of the glial barrier to prevent the extension of the injury site is limited depending on complicated factors, such as the severity of the brain injury, immunity, or the health of the neuron supporting cells. Astrocytes have been proved to synthesize neurotrophic factors (NTFs), which affect the survival, migration, and growth of neurons (Gray and Patel, 1992; Schwartz and Nishiyama, 1994). This involved the reaction of the endogenous neuroprotective system in lesioned brain tissue (Nam et al., 2015; Poyhonen et al., 2019; Jeon et al., 2020). We observed that GFAP^+^ expression was elevated among the penumbra region 7 days after AUDA administration. We suggested that in addition to anti-inflammation and neovascularization, AUDA may enhance the restoration capability of astrocytes in the glial barrier. Therefore, the aggregative astrocytes lead to the increase of NTFs, thereby supporting neuron growth and survival rate in the ischemic penumbra.

## Conclusion

The present study demonstrated the possible therapeutic effects of AUDA in the acute phase of PTI stroke through continuous observation with a lab-developed multimodal optical imaging system. We showed that AUDA treatment reduced the penumbra size and prevented the ischemic core growth. After AUDA treatment, an anti-inflammation reaction possibly occurred at the earliest, thereby playing a role in protecting the neuron from the beginning of the ischemic stroke. The development of neovascularization has been observed 3 days apart, which recovered blood flow and might contribute to ischemic neuron rescue. Furthermore, we found an increased number of astrocytes in the ischemic penumbra following AUDA administration, which may support the role of astrocytes in the anti-inflammation of AUDA.

## Limitations of the Research

There are some potential limitations of this study. To evaluate the consistency of our lab-designed cranial window, we observed the gross changes by monitoring the FVD for over 4 weeks. However, the result may indirectly present the influence of inflammation in a cranial window. The optimal method to examine the anti-inflammation reaction was histological staining using inflammatory markers in uninjured rats. Although such an experiment was not conducted in the present study, we intend to verify the validity of cranial window setup for the long term in our future studies. The other limitation in this study was that a person other than the researchers was not specifically assigned to collect experimental data or analyze it. The absence of blinding may unintentionally lead to the overestimated treatment effect. Therefore, we will adopt blinding during different experiment stages, following the ARRIVE guidelines to reduce analysis bias in our future studies.

## Data Availability Statement

The raw data supporting the conclusions of this article will be made available by the authors, without undue reservation.

## Ethics Statement

The animal study was reviewed and approved by National Yang Ming University.

## Author Contributions

H-LW, J-WC, S-HY, and Y-YC: conceptualization. H-LW, J-WC, S-HY, S-HL, and Y-YC: methodology. H-LW, J-WC, Y-WL, Y-TK, and C-YC: software. H-LW, J-WC, S-HL, and Y-CLi: validation. H-LW, J-WC, S-HY, and Y-YC: formal analysis. H-LW, JWC, S-HY, and Y-YC: investigation. H-LW, J-WC, and H-CP: resources. H-LW, J-WC, and C-YC: data curation. H-LW and J-WC: writing—original draft preparation. H-LW, J-WC, S-HY, H-CP, C-FW, S-HL, Y-CLo, and Y-YC: writing—review and editing. H-LW, J-WC, C-YC, Y-TK, and Y-CLo: visualization. S-HY and Y-YC: supervision. S-HY, S-HL, and Y-YC: project administration. S-HY, S-HL, and Y-YC: funding acquisition. All authors have read and agreed to the published version of the manuscript.

## Conflict of Interest

The authors declare that the research was conducted in the absence of any commercial or financial relationships that could be construed as a potential conflict of interest.
