# Supplementary material for: Multimodal Optical Imaging to Investigate Spatiotemporal Changes in Cerebrovascular Function in AUDA Treatment of Acute Ischemic Stroke
Source: Front Cell Neurosci. 2021 Jun 3;15:655305. doi: 10.3389/fncel.2021.655305 (PMC8209306; doi:10.3389/fncel.2021.655305)
Supplement: Supplementary file 1 [file Data_Sheet_1.docx]

Supplementary Material

Multimodal optical imaging to investigate spatiotemporal changes in cerebrovascular function in AUDA treatment of acute ischemic stroke

***Note 1. In vitro flow phantom experiment to quantitate the relationship between speckle contrast data and true blood flow***

[**Figure S1**](#Figures1) showed the schematic diagram of the experimental setup. The simple straight polyethylene (PE) tubing phantom with actual blood injection mimics the blood flow through a blood vessel, and was connected to a pump by tubing, which was used to validate the changes in speckle contrast acquired by our lab-designed multimodal optical image system with different flow rates. The flow phantom was configured with two glass syringes individually connected to an either end of a 15-cm length of PE-10 tubing filled with actual blood. The blood flow rate in the PE-10 tube was set by adjusting the driving speed of the two-syringe push-pull pump (SP120, World Precision Instruments, Sarasota, FL, USA).

**
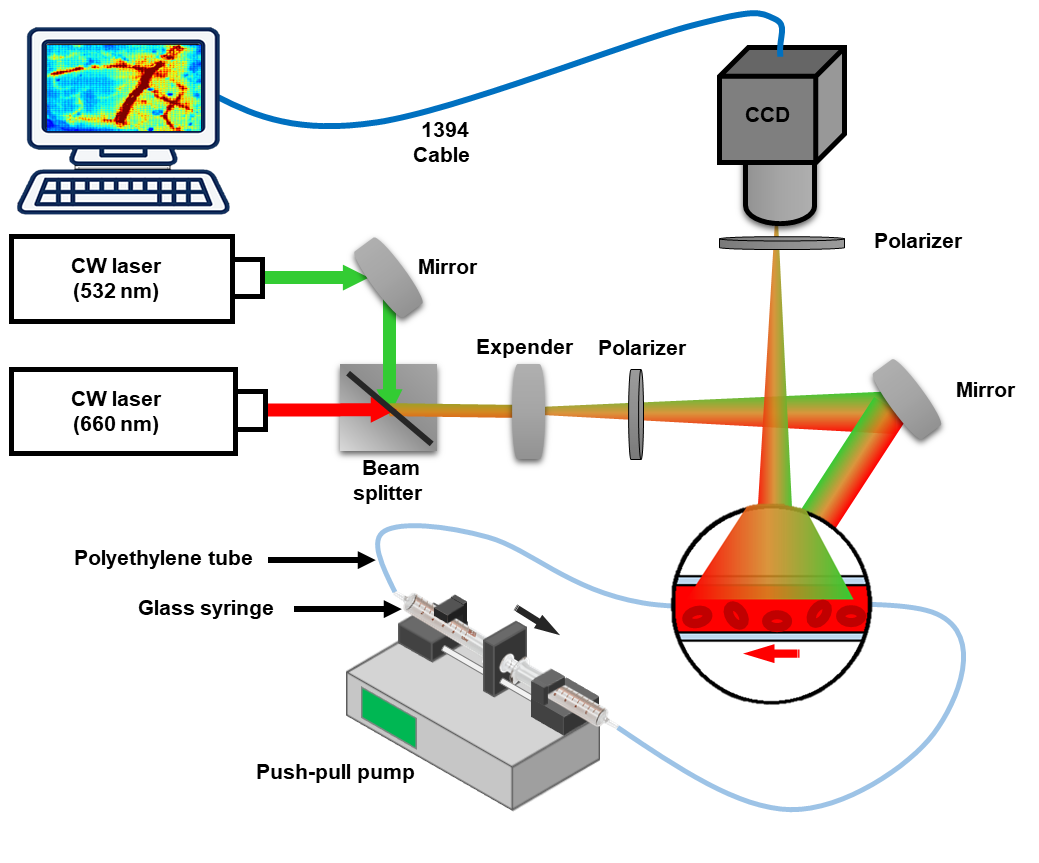
**

**Figure S1. Schematic diagram of the flow phantom experimental setup.** The flow velocity measurement was obtained using LSCI images of rat blood infusing through the PE-10 tubing at flow rates ranging from 0.03 mm/s to 63.11 mm/s, achieved by adjusting the driving speed of the two-syringe push-pull pump.

Before collecting a blood sample, two syringes and the PE-10 tubing were heparinized by aspirating and expelling heparin saline solution (1,000 USP units/mL, Nang Kuang Pharmaceutical Co., Ltd., Tainan, Taiwan) to prevent the formation of blood clots in the flow velocity measurement experiment. Following completion, venous blood was drawn from a rat tail vein using one glass syringe (27 G × 1/2 in., 1.0 mL), and the push-pull pump was used to infuse the animal blood at flow rates ranging from 0.03 mm/s to 63.11 mm/s, which correlated to changes in the speckle flow index (*K*^2^-maps) acquired by our multimodal optical imaging system. LSCI imaging was performed on PE-10 tubing for each blood flow velocities for 10 repetitions. Each scan was acquired for 30 s at frame rates of 15 Hz. The average *K*^2^ value was calculated within the regions of interest (ROI) of 0.2 mm × 3 mm on the PE-10 tubing for each frame and was then averaged over all frames. The correlation between *K*^2^ and actual flow rate was confirmed by least-square regression and Pearson’s correlation analysis.

The result of *K*^2^ against actual flow rate in the flow phantom is shown in [**Figure S2**](#Figures2). We found that the *K^2^* decayed exponentially at lower flow velocities and approximated to saturation (~0) at higher flow velocities (>37.86 mm/sec), as predicted by speckle theory ([Boas and Dunn, 2010](#_ENREF_2)). In addition, *K^2^* exhibited excellent linearity against the actual blood flow velocities from 0.10 mm/s to 5.66 mm/s**,** demonstrating that *K*^2^ was in inverse proportion to the blood flow velocity with the Pearson’s *r =* −0.993 and the corresponding *R^2^* value for a linear fit with a slope of 0.977. Therefore, the linearity of blood flow dynamics provided by our lab-designed multimodal optical imaging system covered the full span of cerebral blood flow velocities in rodent studies ([Autio et al., 2011](#_ENREF_1); [Sato et al., 2017](#_ENREF_4)).


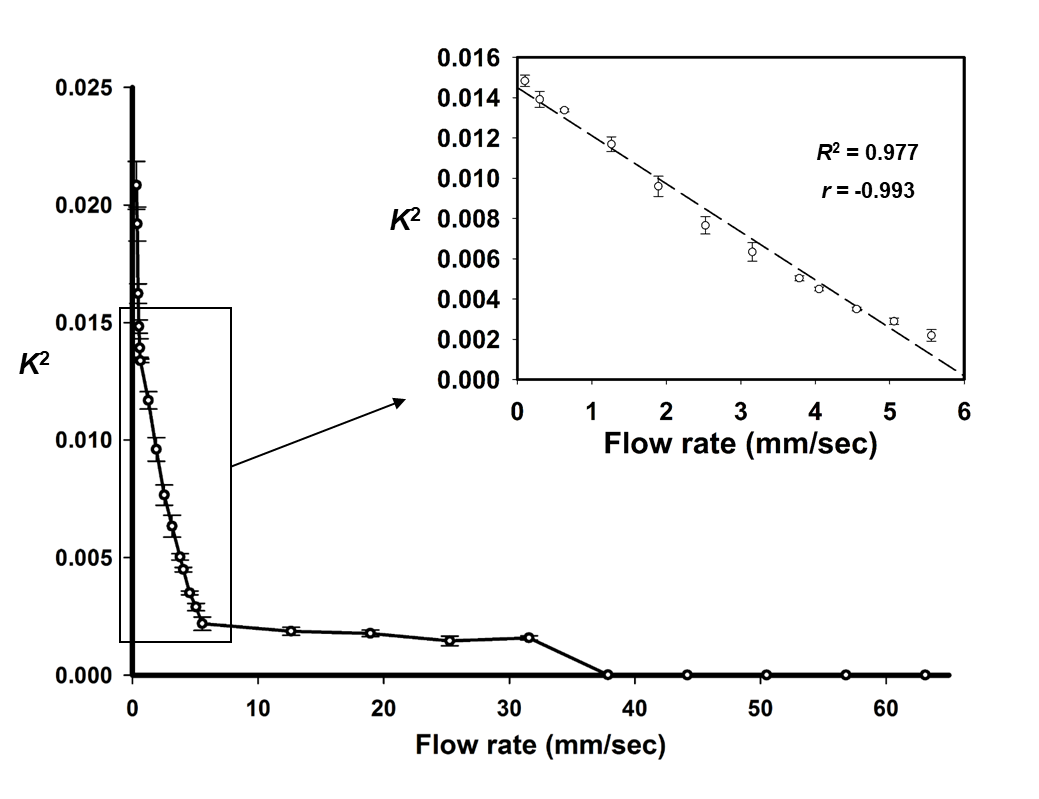


**Figure S2. The flow index (*K*^2^) *vs.* different true flow velocities.** The upper right panel showed that the *K*^2^ is linearly correlated with true flow rates in the range of 0.10 to 5.66 m/s. The Pearson’s *r* = −0.993, *R*^2^ = 0.977. Each point is the averaged *K*^2^ with error bars, presented as the mean ± SEM. *N* = 10.

***Note 2. Evaluation of a lab-designed cranial window with functional vascular density measurement***

To assess the cranial window quality for stable monitoring of long-term structural and functional changes in the brain vasculature, *in vivo* cranial windows were implemented. Thereafter, imaging with our multimodal optical imaging system was conducted on 5 healthy rats immediately after cranioplasty procedure, and follow-up imaging was performed for 28 days. The acquired speckle images were subsequently processed to determine the laser speckle contrast by speckle theory, which was averaged over 10 consecutive images on each recording day ([**Figure S3A**](#Figures3)). Following the conversion of the images to *K*-map, the skeletonized vessel images were reconstructed ([Maragos and Schafer, 1986](#_ENREF_3)), and quantitative image analysis was performed using the functional vascular density (FVD) method ([White et al., 2011](#_ENREF_5)).

The FVD was calculated by dividing the pixels of all skeletonized vessel segments by the total pixels of the cortex area through the cranial window, as shown in **Eq. S1**.

| $FVD= \frac{C}{n}$ | (S1) |
| --- | --- |

where C denotes the total number of pixels of the skeletonized vessel, and n denotes the total number of pixels in the skeletonized image. To assess the changes in vascular density over the time course, the percentage change of *FVD* (*∆FVD %*) was calculated by dividing the *FVD* value with the value on day 0 as follows:

| $\Delta FVD \%= \frac{{FVD}_{t}-{FVD}_{0}}{{FVD}_{0}}$ | (S2) |
| --- | --- |

where *FVD_0_* denotes the *FVD* on day 0 and *FVD_t_* denotes the *FVD* on *t^th^* day with *t* = 0, 1, 3, 5, 7, 10, 13, 16, 19, 22, 25, and 28.

[**Figure S3A**](#Figures3) shows representative speckle contrast images on observation days and the corresponding skeletonized images are shown in [**Figure S3B**](#Figures3). There was no serious loss of vascularity observed, and the optical clarity of the window was maintained since implantation of the lab-designed cranial window, suggesting that no brain inﬂammation occurred ([**Figure S3A**](#Figures3)). We further quantified the changes in vascular density as *∆FVD %* within the imaging windows and analyzed the data using a one-way ANOVA followed by Tukey *post-hoc* test (*N* = 5). There were no significant changes in vascular morphology after the implantation of the cranial window over 28 days compared with those of day 0 (**Figure S4**).

**
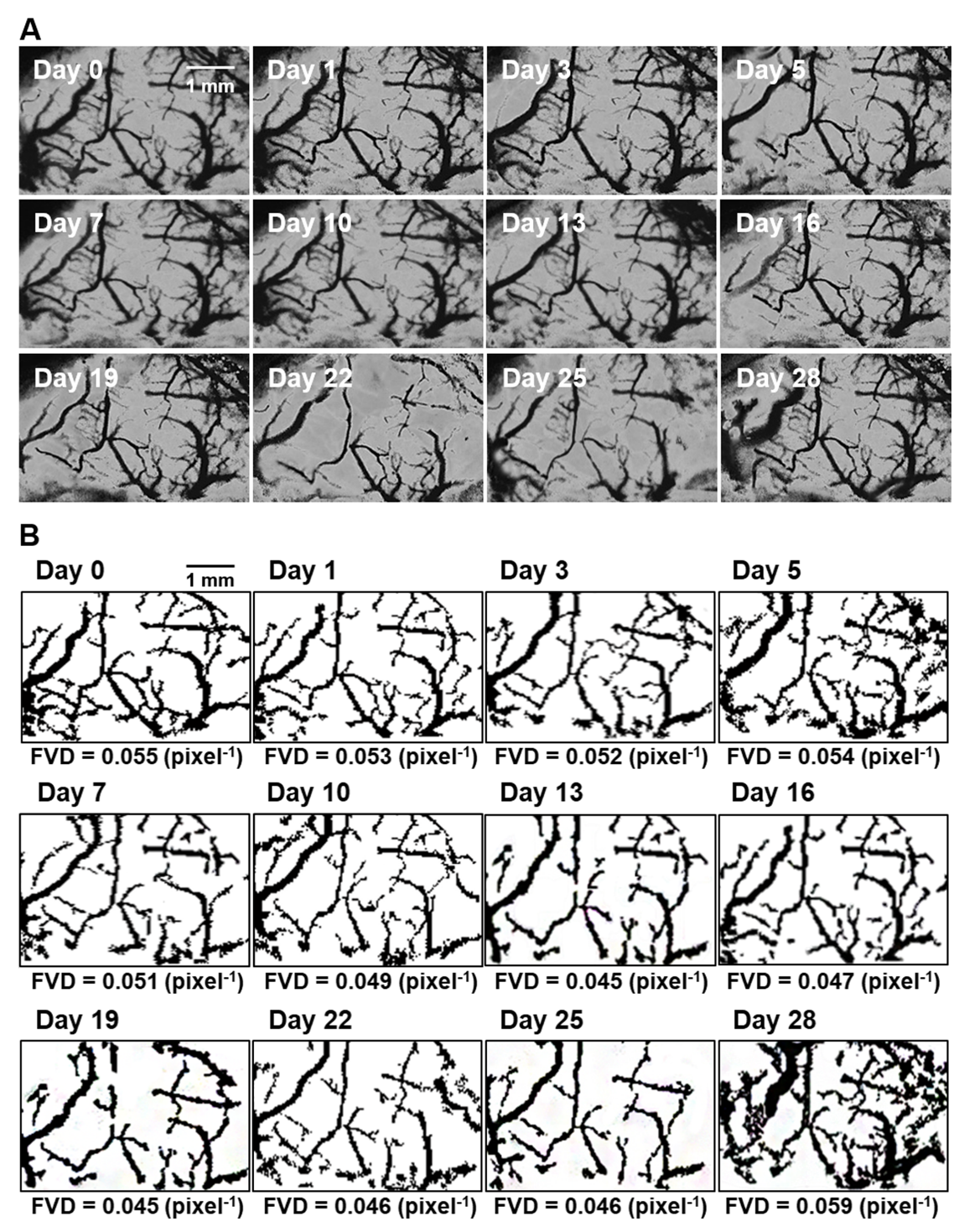
**

**Figure S3. *In vivo* speckle contrast images and their corresponding skeletonized vessel images for quantification of changes of FVD over 28 days.** (a) Long-term *in vivo* LSCI images from a rat bearing the cranial window. (b) Skeletonized images converted from corresponding speckle contrast images in (a). The skeletonized vessels were quantitated with the FVD method. The scale bar is 1 mm.


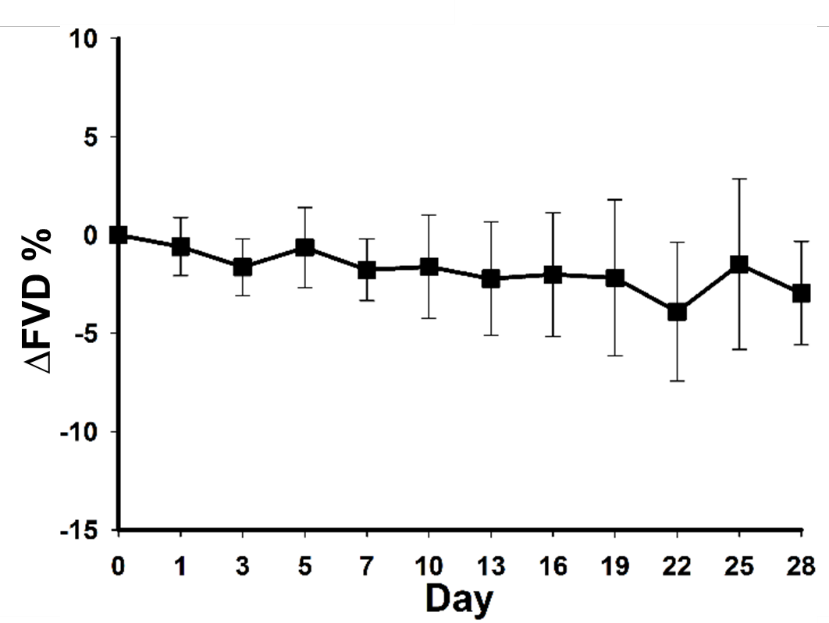


**Figure S4. ∆*FVD* % over 28 days. N**o significant differences were observed in ∆*FVD* % following implantation of the lab-designed cranial window over 28 days. (One-way ANOVA followed by Tukey *post-hoc* test, *N* = 5). Data are presented as the mean ± SEM.

***Note 3. Hemoglobin dynamic changes in the presence of forepaw electrical stimulation***

As shown in **Figure S5**, the representative images of hemoglobin oxygenation showed the spatial maps in stimulus-induced maximal ∆[HbO], ∆[Hb], and ∆[HbT] over days. The ∆[HbO] in the AUDA-treated animal were the increases of 7.17 ± 2.36 μM, 8.58 ± 2.23 μM, 16.84 ± 2.15 μM, and 22.87 ± 2.31 μM at 1, 3, 5, and 7 days after stroke, respectively, which were higher than those of the control animal in the S1FL area. Furthermore, the ∆[Hb] in the AUDA-treated animal were lower than those in the control group at 1, 3, 5, and 7 days post-stroke, with the decreases of -7.81 ± 2.30 μM, -8.58 ± 2.47 μM, -15.51 ± 1.84 μM, and -20.25 ± 1.94 μM, respectively.

**Figure S6** also showed the time course measurements of hemoglobin changes in control group and AUDA-treated group. During 20-sec stimulation, the increased ∆[HbO], ∆[Hb], and ∆[HbT] were observed in pre-stroke (pre) and post-stroke (day 0) status in both control and AUDA-treated groups. However, evoked ∆[HbO] in the AUDA-treated group (day 1: 4.98 ± 1.16 μM; day 3: 14.57 ± 1.04 μM; day 5: 19.74 ± 2.16 μM; day 7: 9.81 ± 0.95 μM) was higher than those in the control group (day 1: 4.14 ± 1.58 μM; day 3: 0.87 ± 0.91 μM; day 5: 2.23 ± 1.66 μM; day 7: 2.90 ± 1.14 μM). Similarly, the increased ∆[HbT] in the AUDA-treated group (day1: 4.28 ± 1.74 μM; day 3: 10.11 ± 1.64 μM; day 5: 12.82 ± 2.05 μM; day 7: 13.84 ± 1.64 μM) was also higher than those in the control group (day 1: 2.01 ± 1.05 μM; day 3: 0.32 ± 0.20 μM; day 5: 2.02 ± 1.54 μM; day 7: 2.68 ± 1.33 μM). Values for ∆[Hb] in the AUDA-treated group (day1: -4.55 ± 1.65 μM; day 3: -4.64 ± 1.38 μM; day 5: -9.44 ± 1.73 μM; day 7: -9.66 ± 1.25 μM) was lower than those in the the control groups (day 1: -3.13 ± 1.47 μM; day 3: -1.12 ± 0.84 μM; day 5: -0.08 ± 0.47 μM; day 7: -2.01 ± 1.26 μM).

**
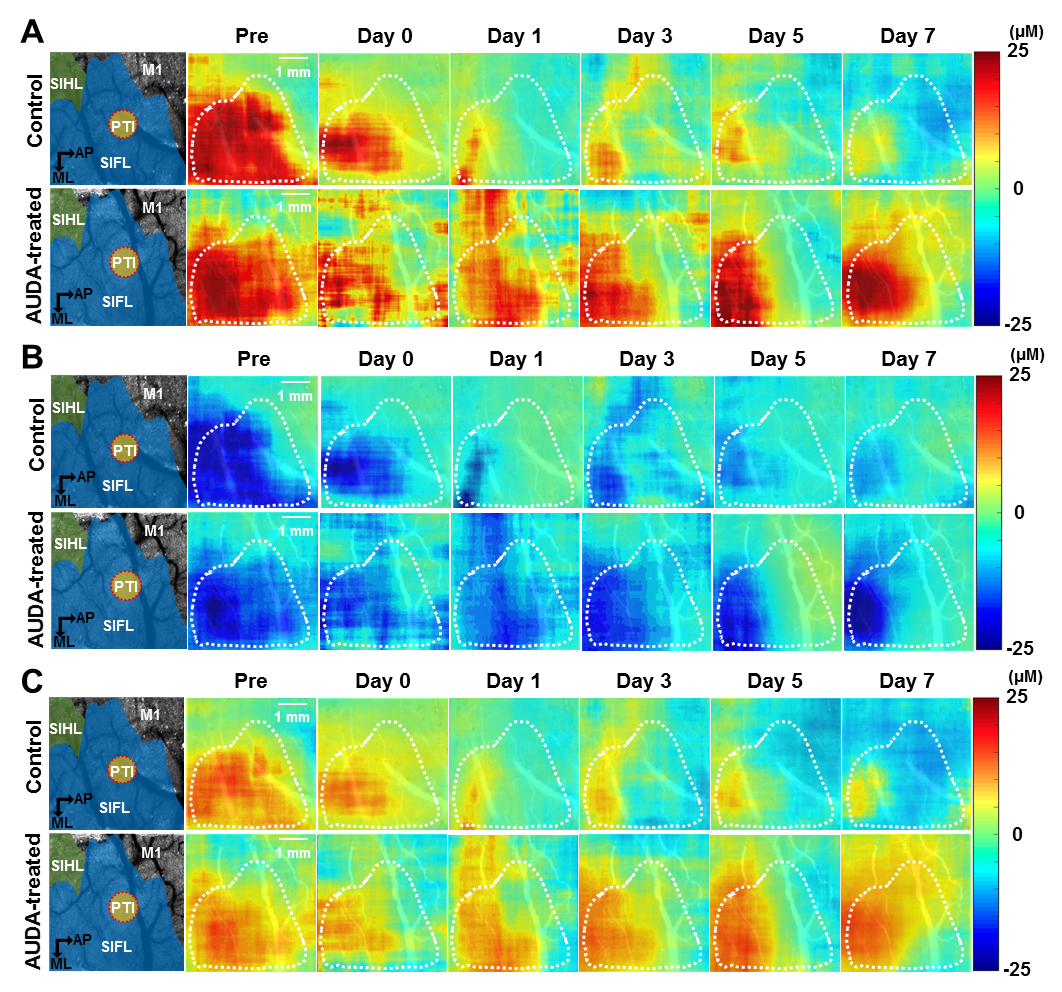
**

**Figure S5. Comparison of stimulus-evoked changes in hemoglobin oxygenation in the rat brain cortical areas between sham control and AUDA treatment.** The spatio-temporal maps of (A) ∆[HbO], (B) ∆[Hb], and (C) ∆[HbT] in concentration (μM) were shown for two representative rats individually belonging to control and AUDA-treated groups. Compared to the control group, gradual recovery of ∆[HbO], ∆[Hb], and ∆[HbT] following PTI stroke were found in the AUDA -treated animal.


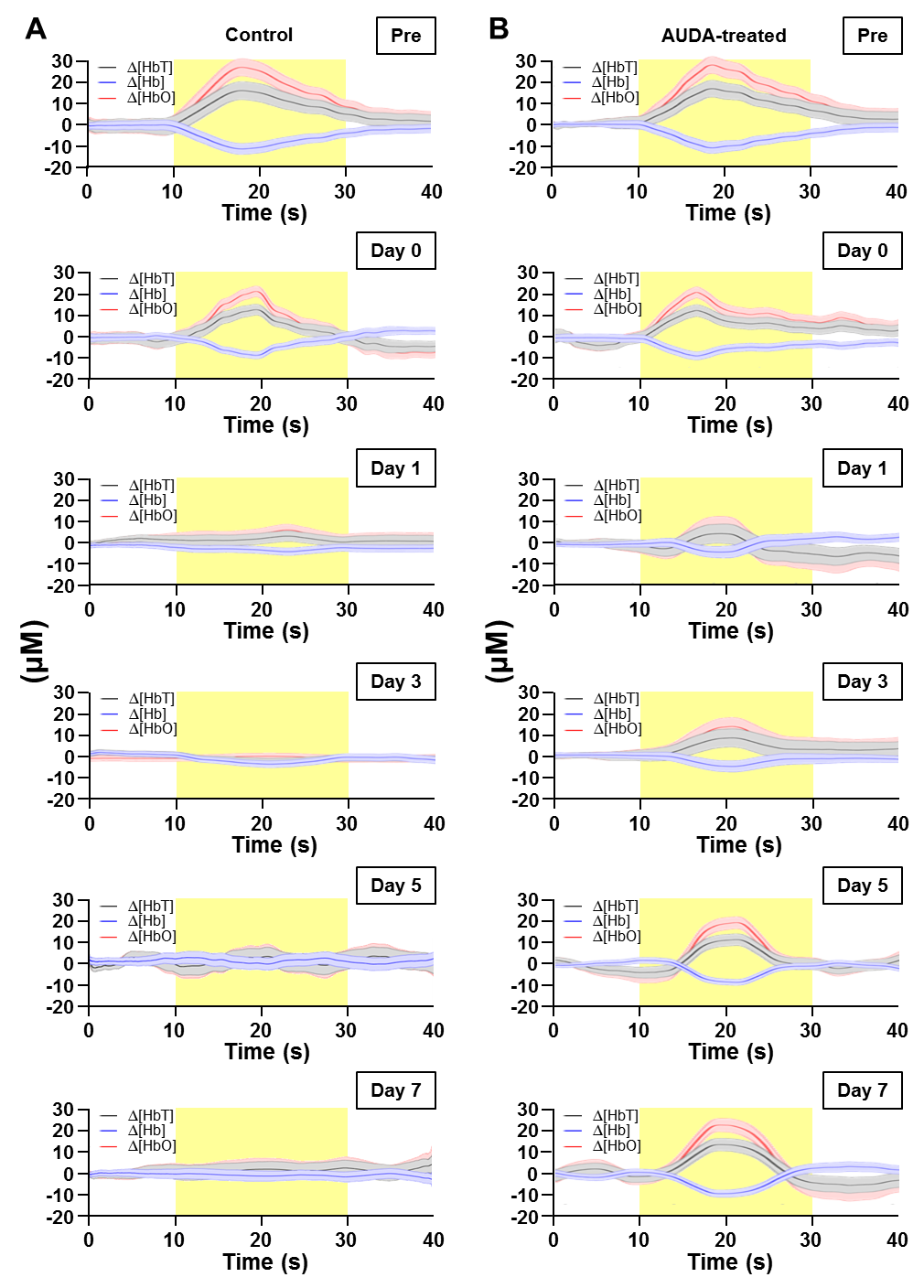


**Figure S6. Group analysis of the time course of ∆[HbO], ∆[Hb], and ∆[HbT] in the S1FL area.** (A) There were no obviously stimulus-evoked hemodynamic responses found in the control group from post-stroke day 1 to day 7. (B) The gradual increases in stimulus-evoked hemodynamic responses were observed over time following PTI stroke in the AUDA-treated group. The yellow box represented the period of forepaw stimulation. The shadow error bar indicated the SEM among the subjects (*N* = 10 for each group).

**References**

Autio, J., Kawaguchi, H., Saito, S., Aoki, I., Obata, T., Masamoto, K., et al. (2011). Spatial frequency-based analysis of mean red blood cell speed in single microvessels: investigation of microvascular perfusion in rat cerebral cortex. *PLoS One* 6, e24056. doi: 10.1371/journal.pone.0024056

Boas, D.A., and Dunn, A.K. (2010). Laser speckle contrast imaging in biomedical optics. *J Biomed Opt* 15, 011109. doi: 10.1117/1.3285504

Maragos, P., and Schafer, R. (1986). Morphological skeleton representation and coding of binary images. *IEEE Trans Acoust Speech Signal Process* 34, 1228-1244. doi: 10.1109/ICASSP.1984.1172472

Sato, T., Dejima, H., Haruta, M., Kamikawa, S., Nakazawa, H., Tokuda, T., et al. (2017). Automatic Determination of Blood Flow Velocity in Brain Microvessels in a Cerebral Infarction Model Mouse Using a Small Implantable CMOS Imaging Device. *Adv Biomed Eng* 6, 68-75. doi: 10.14326/abe.6.68

White, S.M., George, S.C., and Choi, B. (2011). Automated computation of functional vascular density using laser speckle imaging in a rodent window chamber model. *Microvasc Res* 82, 92-95. doi: 10.1016/j.mvr.2011.03.006
